# Dietary Antioxidants and Prevention of Non-Communicable Diseases

**DOI:** 10.3390/antiox7070094

**Published:** 2018-07-19

**Authors:** Giuseppe Grosso

**Affiliations:** NNEdPro Global Centre for Nutrition and Health, St John’s Innovation Centre, Cambridge CB4 0WS, UK; g.grosso@nnedpro.org.uk; Tel.: +39-095-378-2182

According to the recent report of the World Health Organization (WHO), the global burden of non-communicable diseases (NCDs) has been rising over the last century, with the leading causes of disability being depression, diabetes, cardiovascular diseases (CVDs), and certain cancers [1]. Besides genetic, environmental, and socioeconomic factors, exploring dietary factors influencing these conditions became of primary importance in order to better define effective strategies for reducing the burden of disease [2]. In fact, higher adherence to healthy and equilibrated dietary patterns has been shown to be implicated in prevention of NCDs [3].

Numerous epidemiological studies have demonstrated the association between oxidative stress and NCDs. Oxidative stress is commonly known as an imbalance in the production of reactive oxygen species (ROS) and the biological antioxidant defense system. Over the last years, exogenous antioxidants have received great attention because of their potential beneficial effects toward human health. Contained in foods commonly consumed in all populations worldwide, antioxidants represent an attractive explanation of their beneficial effects. However, antioxidants are contained not only of fruits and vegetables, which are characteristic components of healthy dietary patterns, but also in other plant-derived foods, such as tea, coffee, and cocoa. Therefore, the evaluation of dietary habits of a population, and in particular the intake of antioxidants and adherence to healthy dietary patterns is crucial. Indeed, several studies explored the dietary intake of antioxidants and antioxidant-rich foods in a Mediterranean area, as well as their association with fluid and beverage intake [4,5,6], demonstrating that relatively healthy dietary habits are common in southern Italy.

Accumulating epidemiological evidence have demonstrated the association between both antioxidants and antioxidant-rich foods intake and human health. For instance, recent meta-analysis and cohort studies showed that high consumption of antioxidants and antioxidant-rich foods is associated with decreased risk of overall and CVD-related mortality [7], certain cancers [8], CVD [9], and mood disorders [10]. Importantly, numerous reviews summarized and highlighted the preventive role of antioxidants contained in grape seeds [11], cocoa [12], *Moringa oleifera* leaves [13], coffee [14], mulberry fruit [15], and brown rice [16].

Nevertheless, nowadays studies exploring the possible effects of antioxidants toward human health, should take into consideration the differences in dietary intake of polyphenols in various populations, differences in food processing (loss of phenolic content), absorption, bioavailability, and metabolism of polyphenols. The study by Khairallah et al. focused on the antioxidant effect of phenolic extracts from polyphenol-rich potato, demonstrating that the colonic microbial digestion of potato-based polyphenols could lead to improved colonic health, as this process generates phenolic metabolites with significant antioxidant potential [17].

Several molecular mechanisms may account for the beneficial effects of polyphenols. The antioxidant effects of dietary polyphenols can be attributed to the regulation of redox enzymes through reducing reactive oxygen species (ROS) production and modulation of the II-phase enzymes responsible for the cellular oxidative response. Indeed, the study by Marmouzi et al. showed that antioxidant compounds from hybrid oat lines prevent against hyperglycemia-induced oxidative stress via the modulation of expression of key II-phase enzymes [18]. Additionally, antioxidants may exert chemo-preventive effects through a variety of mechanisms, including the elimination of carcinogenic agents, the modulation of pathways responsible for cancer cell signaling and cell cycle progression, and by the promotion of apoptosis. A review by Losada-Echeberría et al. summarized the evidence of the antitumor effects of plant polyphenols on breast cancer, with special attention to their activity on estrogen receptors (ERs) and human epidermal growth factor receptor 2 (HER2) targets and also covering different aspects, such as redox balance, uncontrolled proliferation, and chronic inflammation [19].

Further evidence from both epidemiological and experimental studies is needed in order to better characterize antioxidants that may exert beneficial effects toward the prevention of chronic diseases associated with oxidative stress and inflammation.
